# Supplementary material for: Evaluation of host effects on ectomycorrhizal fungal community compositions in a forested landscape in northern Japan
Source: R Soc Open Sci. 2020 Feb 19;7(2):191952. doi: 10.1098/rsos.191952 (PMC7062096; doi:10.1098/rsos.191952)
Supplement: Supporting Information Figures [file rsos191952supp1.docx]

***Supporting Information***

Article title: Evaluation of host effects on ectomycorrhizal fungal community compositions in a forest landscape in northern Japan

Authors: Shunsuke Matsuoka, Yoriko Sugiyama, Ryunosuke Tateno, Shihomi Imamura, Eri Kawaguchi, Takashi Osono.

The following Supporting Information is available for this article:

Fig. S1 Rarefaction curve for each soil sample (n = 180).

Fig. S2 Community dissimilarity among the plots as revealed by nonmetric multidimensional scaling (NMDS) ordination using the Raup-Crick index (stress value = 0.132). Plot numbers in the symbols are consistent with those listed in Table 1 and Fig. 1.

Fig. S3 Venn diagram showing the effects of host species and spatial distance on the ectomycorrhizal (ECM) fungal community composition as derived from the variation partitioning analysis using the Raup-Crick index. Numbers indicate the proportions of explained variation. No shared fraction between the host species and spatial variables was detected.

Fig. S4 Family level proportions of ECM fungal OTU for each host tree species.


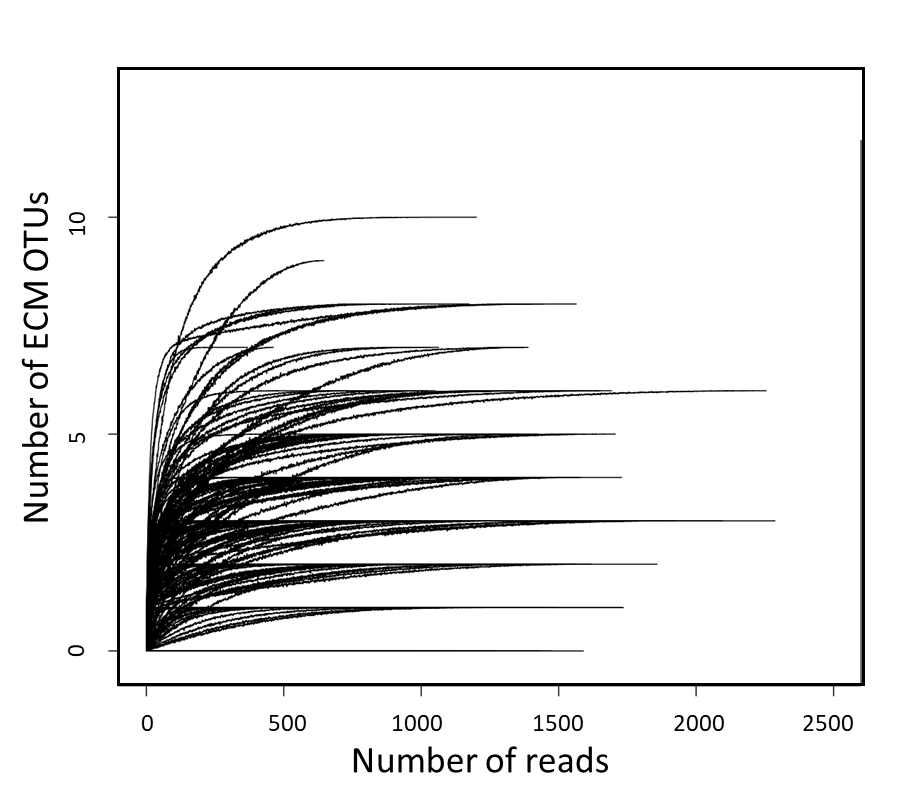


Fig. S1 Rarefaction curve for each soil sample (n = 180).


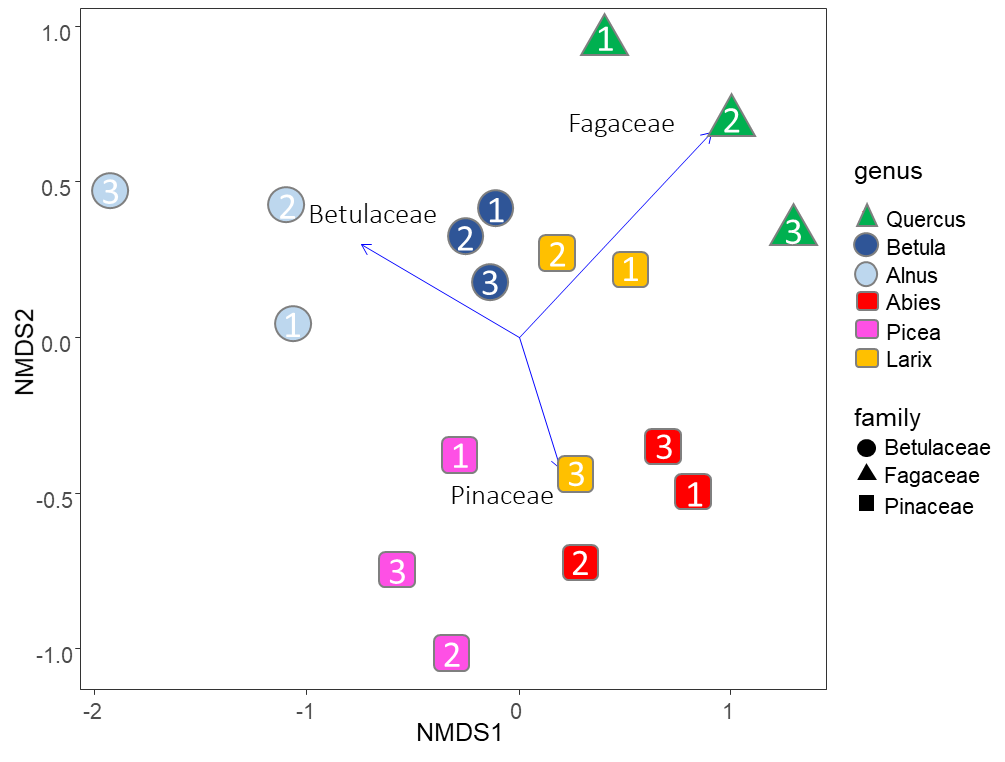


Fig. 2 Community dissimilarity among the plots as revealed by nonmetric multidimensional scaling (NMDS) ordination using the Raup-Crick index (stress value = 0.132). Plot numbers in the symbols are consistent with those listed in Table 1 and Fig. 1.


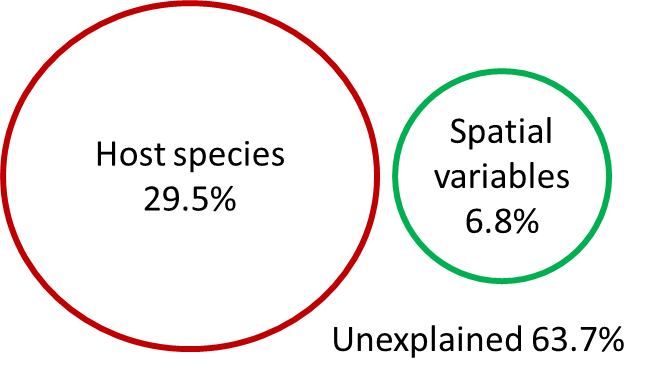


Fig. 3 Venn diagram showing the effects of host species and spatial distance on the ectomycorrhizal (ECM) fungal community composition as derived from the variation partitioning analysis using the Raup-Crick index. Numbers indicate the proportions of explained variation. No shared fraction between the host species and spatial variables was detected.


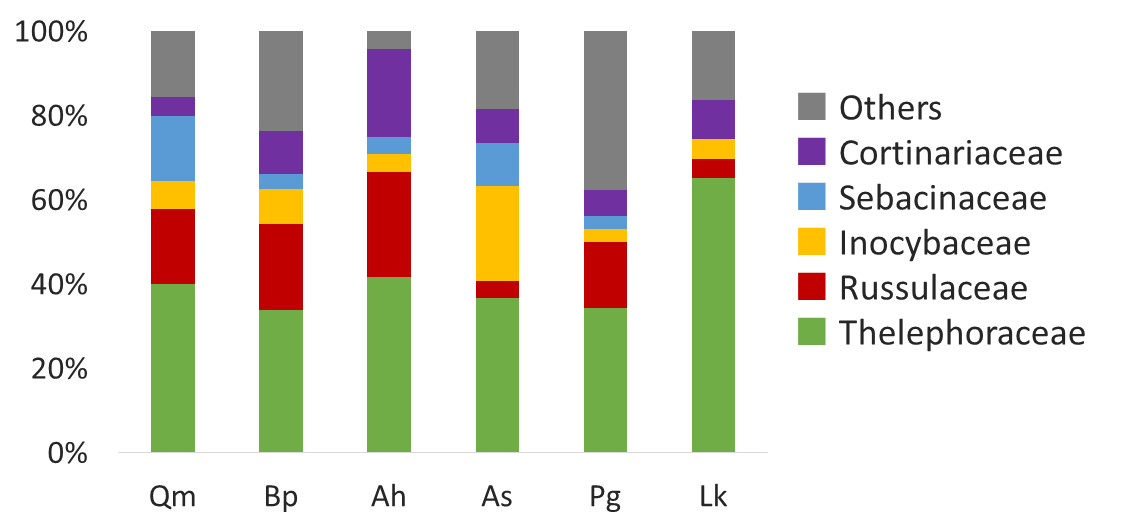


Fig. S4 Family level proportions of ECM fungal OTU for each host tree species.
